# Sexuality and cancer in adolescents and young adults - a comparison between reproductive cancer patients and patients with non-reproductive cancer

**DOI:** 10.1186/s12885-019-6009-2

**Published:** 2019-08-22

**Authors:** Julian Mütsch, Michael Friedrich, Katja Leuteritz, Annekathrin Sender, Kristina Geue, Anja Hilbert, Yve Stöbel-Richter

**Affiliations:** 1 Department of Mental Health, Medical Psychology and Medical Sociology, University of Leipzig, Philipp-Rosenthal-Str. 55, 04103 Leipzig, Germany; 2 Faculty of Managerial and Cultural Studies, University of Applied Sciences Zittau / Goerlitz, P. O. Box 30 06 48, 02811 Goerlitz, Germany; 3 Departments of Medical Psychology and Medical Sociology and Psychosomatic Medicine and Psychotherapy, Integrated Research and Treatment Center AdiposityDiseases, Behavioral Medicine, University of Leipzig, Philipp-Rosenthal-Str. 27, 04103 Leipzig, Germany

**Keywords:** Adolescents and young adults, AYA, Cancer, Sexuality, Reproductive cancers, Non-reproductive cancers, Quality of life

## Abstract

**Background:**

Sexuality is an important aspect of quality of life for adolescent and young adults that remains understudied in cancer patients. Most current knowledge about how cancer and cancer treatments can affect patients’ sexuality pertains to reproductive cancer patients (breast, gynecological, male reproductive organs), whereas only little is known about how the disease affects the sex lives of patients with other types of cancer. This study examined sexual satisfaction and sexual supportive care needs among adolescent and young adult cancer patients, with a particular focus on how the type of cancer a person has is associated with these issues differently.

**Methods:**

Five hundred seventy-seven (*n* = 424 females, 73.5%) patients between 18 and 39 years of age at diagnosis and representing all major tumor entities completed the standardized questionnaire. The analysis addressed the following topics: sexual satisfaction (Life Satisfaction Questionnaire), sexual supportive care needs (Supportive Care Needs Survey), and changes in sexuality (Questions on Life Satisfaction Modules). These topics were tested by mean differences between reproductive and non-reproductive cancer, equivalence testing and regression analyses.

**Results:**

About one third of the patients reported being dissatisfied with their sexuality and having supportive care needs in this area. Changes in sexuality were significantly more common in women with reproductive cancers than in those who had other types of cancer (*t* = − 2.693, *p* = .007), while both groups had equivalence in scores for sexual satisfaction and sexual supportive care needs. Reproductive cancers are not more associated with deterioration of sexual satisfaction (*R*^*2*^ = .002, *p* = .243), changes in sexuality (*R*^2^ = .006, *p =* .070) or increased sexual supportive care needs than non-reproductive cancers (*R*^*2*^ = .004, *p* = .131).

**Conclusions:**

The results indicate that about a third of adolescents and young adults with both reproductive but also with non-reproductive cancer experience sexual dissatisfaction in similar measure. An equal percentage of these patients also express a desire to receive supportive care in this area. Consequently, health care professionals should address issues of sexuality and cancer as a matter of routine when caring for young adults even when patients have a non-reproductive cancer.

**Electronic supplementary material:**

The online version of this article (10.1186/s12885-019-6009-2) contains supplementary material, which is available to authorized users.

## Background

The National Cancer Institute defines patients between 15 and 39 years old as adolescents and young adults (AYA) [1], a population characterized by the significant physical, social, emotional and cognitive developments people undergo at this formative stage of life [2]. Sexuality plays a central role in developing into an adult and is an important aspect of well-being and quality of life [3]. The process of sexual maturation is a deep physical and psychological change that affects the course of a person’s entire life. During adolescence primary and secondary sexual characteristics begin to develop and the experience of both having and being the object of sexual desire evolves [4]. Often it also a time during which a person’s first sexual interactions take place, while early adulthood is marked by gaining sexual experience [5, 6]. And although most sexual intercourse during emerging adulthood takes place in the context of relationships, casual sex is also common [6]. Young adults’ sexuality has a significant impact on the rest of their lives as that is usually the time during which long-term partnerships are formed and family planning takes place [7].

In 2013, about 15,500 Adolescents and Young Adults in Germany were diagnosed with cancer. Of these, 14,000 were between the ages of 18 and 39 (database request from [8]). This populations’ overall survival rate of about 80% 15 years post-treatment is well above average in Germany [9]. The body of research that exists on AYA with cancer has consistently shown that they have specific issues and needs that set them apart from both younger children and older adult cancer patients [10, 11]. A cancer diagnosis can substantially threaten important developmental processes that take place during adolescence and early adulthood. Not only are these patients tasked with the challenges everyone has in becoming independent adults, they are also simultaneously confronted with a potentially life threatening disease which can entail: having to deal with physical and psychological changes; having to be more dependent on others (professional medical staff, parents); difficulties in their social lives; missing school, apprenticeship or work due to treatment; and the potential loss of fertility, all of which can be sources of overall distress [12, 13]. Other problems that can arise when coping with the disease are: changes in body image, depression, anxiety, somatization, and partnership problems [14–16].

Cancer can threaten a person’s sexual identity in a variety of ways [17–19]. Not only do the disease and its treatment often cause physical changes [20, 21] and affect fertility [22] they can also impose psychological burdens that impact patients’ self-esteem [23], sexuality, and partnerships [24]. Issues that women specifically face can include: early onset of menopause [25], dyspareunia [26], lubrication problems [27], and vaginal stenosis [28], while men are vulnerable to erectile dysfunction [29]. Both sexes are affected by: loss of satisfaction [30], orgasmic problems [31], loss of desire and libido [32, 33], fatigue [34] and infertility [21, 23]. These are mostly general findings but certain research indicates that some of these problems affect AYA patients differently. A recent review done by Warner et al. [35] concluded that AYA cancer patients and survivors lack pertinent knowledge concerning how the disease can affect their sexuality. After comparing younger and older breast cancer survivors with an age-matched control, Champion et al. [36] showed that younger breast cancer survivors had worse sexual functioning than the age-matched control and older survivors. This clearly indicates that AYA patients seem to have a more difficult time dealing with cancer. Bellizzi et al. [12] reported that AYA patients with germ cell, lymphatic, and haematological cancers as well as sarcomas often complained that the illness had a significant negative impact on their sexual functioning and intimate relationships. They also pointed out that this important topic is understudied.

Most of the research that has been done on sexuality among cancer patients has focused on cancers that directly affect sexual organs (testicles, uterus, ovaries, mama, prostate, penis = reproductive cancer = RC) [37]. However, it is evident that sexuality is also impaired in patients who have types of cancer that originate in other parts of the body such as: colorectal [38], head and neck [39], and haematological cancers [40] (all other diagnoses = Non-reproductive cancer = NRC). Perz et al. [41], who conducted a study with a sample outside of the AYA age range, found that NRC patients were more likely to rate sexuality as being important to them than patients with reproductive cancers (RC) were. Greenfield et al. [42] analysed 25–45 years old males with a variety of cancer diagnoses and detected lower sexual drive, arousal, activity, orgasm quality, and general sexual functioning than in healthy controls.

In this context, the aim of this study was to broaden the general base of knowledge about sexuality in AYA cancer patients as well as to compare the sexuality of RC patients, which has thus far received more scientific attention, to that of patients with other types of cancers. Our hypothesis was that patients with RCs are less satisfied with their sexuality after treatment, perceive more changes in their sexuality, and need more support in this area than non-reproductive cancer patients.

### Method

The data were derived from the “AYA-LE” study, a prospective longitudinal study with 2 measuring points, which investigated the frequency of psychological distress, and the effect of cancer on quality of life and life satisfaction both in general and in specific life domains among AYA cancer patients. Patients were recruited over a period of 20 months in cooperation with 16 oncological acute care hospitals, two local tumour registries, and four (cancer) rehabilitation clinics specialised in treating AYA patients. Overall, 762 patients received the study information and gave written consent. Of these, 281 (36.9%) patients were recruited by the four rehabilitation clinics, 207 (27.2%) from two tumor registries, 82 (10.8%) from 16 acute hospitals, and 192 (25.2%) patients self-registered. 185 patients were excluded either because they declined to participate (*n* = 43), did not meet the inclusion criteria (*n* = 88), or did not respond (*n* = 54). The study inclusion criteria were: a) age at diagnosis between 18 and 39 years (Adolescents under 18 in Germany are mostly treated in paediatric wards.); and b) first diagnosis of a cancer at any tumour site and diagnosis within the last 48 months. Finally 577 patients entered in the study. Further information about the study design and recruitment can be found at Leuteritz et al. [43]. After consenting to take part in the study (patient master sheet and consent form), participants responded either via online or paper questionnaires that were compiled from several standardized instruments. These instruments, like the European Organisation for Research and Treatment of Cancer Quality of Life Questionnaire (EORTC QLQ C-30), the Hospital Anxiety and Depression Scale (HADS), the Supportive Care Needs Survey Questionnaire (SNCS-SF34-G) and further details about the sample characteristics, are specified in the study protocol [44]. The original questionnaire is added as “Additional file 1” in the supplementary material section. All participants were notified about the research background of the study, the voluntary nature of their participation, and their right to refuse to enrol. In addition to an accompanying official letter from the research team about the research project, a data privacy statement was distributed, which assured the strict confidentiality of all information shared in the questionnaire, and informed participants as to how their personal data would be handled. All of the subjects gave written informed consent and completed several questionnaires. The study procedure was approved by the ethics committee of the University of Leipzig (Reference number 372–13-16,122,013).

## Measures

In addition to sociodemographic (e.g. gender, age, occupation, partnership, children) and medical data (e.g. cancer diagnosis, medical treatments), we also assessed psychological factors (e.g. satisfaction with sexuality and partnership, quality of life, social support) using standardized questionnaires and single items.

### Sexual satisfaction

#### The life satisfaction questionnaire (FLZ) – sexuality scale (FLZ Sex)

This questionnaire is a German instrument designed to evaluate ten different life domains that contribute to life satisfaction. Each of the ten subscales contains seven items that are rated on a 7-point response scale from 1 (*very dissatisfied*) to 7 (*very satisfied*). The item scores are summarized in a total scale score (7 to 49) whereby higher scores indicate a higher level of satisfaction in the following categories: sexual attraction, sexual efficiency, sexual contacts, sexual response, sexual partner interaction, and communication. The internal consistency for the sexuality scale is Cronbach’s α = 0.92 [45]. For this sample, Cronbach’s α for the sexuality scale was α = 0.88.

#### Questions on life satisfaction (FLZ-M)

The FLZ-M is a questionnaire comprised of 8 modules that represent different important areas of life. We used the “*partner relationship and sexuality*” module for the present study. Respondents were asked to rate their level of satisfaction with this part of their life on a scale of 1 (*highly dissatisfied*) to 5 (*extremely satisfied*). The internal consistency of the total FLZ-M score is Cronbach’s α = 0.82 [46]. For this sample, Cronbach’s α was α = 0.84. Our study posed the two-pronged question: “How satisfied are you with the following areas of your life”: “sexuality” and “partnership”. We used the answers of the two questions independently as a scale from 1 to 5 in the analysis.

### Changes in sexuality

#### Questions concerning changes in life satisfaction – (FLZ-MC)

In addition, we asked how much the FLZ-M defined areas of life had changed for the participants since their diagnosis. Responses were given on a 5 - point scale (1 = *not at all* to 5 = *very much*) corresponding to the other modules of the FLZ-M. For this sample, Cronbach’s α for all areas of life was α = 0.83. The question we used was: “*How much has your sexuality changed since you were diagnosed?*”. The answers were used as a scale from 1 to 5 in the analysis.

### Sexual supportive care needs

#### Supportive care needs survey questionnaire (SCNS-SF34-G) – sexuality needs domain (SCNS-SF34 Sex)

This questionnaire includes 34 items and is divided into 5 domains that capture whether patients feel they need support, and if so, how much. The items are rated on a 5-point response scale (1 = *no need* to 5 = *high need*). The sexuality domain is comprised of three items. Standardization of domains from 0 to 100 (higher score = higher level of perceived need) as well as dichotomization of raw scores into “*no need*” vs. “*some need*” is possible. The German version of this subscale has an internal consistency of Cronbach’s α = 0.82 [47]. For this sample, Cronbach’s α was α = 0.75.

## Analysis

The statistical analysis was performed with Statistical Package for Social Sciences 20 (SPSS by IBM) and with Microsoft Excel Version 15.31. We analysed men and women separately because it is known that sexuality differs between the genders and cancer impacts them uniquely [48–50]. Frequency differences were tested with χ^2^-analyses. Student’s t-test was performed to analyse differences between RCs and NRCs regarding satisfaction with sexuality and supportive care needs and Welch’s t-test was used when there were unequal variances. Equivalence testing was performed with two one-sided tests (TOST) according to Lakens et al. [51] using the described spreadsheet Version 0.4.4. Equivalence boundaries for the FLZ Sex scale were set at ±4 raw points. Equivalence was rejected if the mean of one group was 4 points higher (on a 7-point scale with 7 questions) than the mean of the comparison group. These equivalence boundaries seemed appropriate for quantifying a substantial effect when using a questionnaire with a range of 42 points between the lowest and highest possible values and an overall pooled standard deviation of 9.154. An effect of 4 points with an *SD* of 9.154 would be a difference of 0.43 *SD* for women and 0.48 SD for men, slightly lower than a medium effect of Cohen’s *d* = 0.5 [52]. For the SCNS-SF34 sex domain, the values of the 3 items (each with 5 possible points) were converted to a scale of 0–100 whereby equivalence boundaries were set at ±16.67 points, which represented ±2 raw points. This resulted in an effect of *d* = 0.61 for men and *d* = 0.51 for women. For the FLZ-M Sex and FLZ-MC Sex scales (each of which has a range of 5 points), equivalence boundaries were set at ±0.5 raw points. Consequently, they represented effects between *d* = 0.33 and *d* = 0.39. A hierarchical multiple linear regression was performed to measure the impact of reproductive cancers versus non-reproductive cancers on patients’ sexual satisfaction and sexual supportive care needs. Assumptions for multiple regression: normality, linearity, multicollinearity and independence of residuals were checked. P-P plots and histogram for normality, plot of standardized residuals against standardized values to check for linearity and homogeneity of variance. The absence of multicollinearity was controlled by Variance inflation factor statistic and independence of residuals were checked by Durbin-Watson statistic. Prior to regression analysis the FLZ-Sex, FLZM-Sex and FLZ-MC-Sex scores were z-standardized. The first model included factors already known to influence these criterions: gender [48], age [53], and partnership [54]; and the next model added the variation of NRC vs. RC. Post-hoc power analysis was conducted with G*Power 3.1.9.2 [55] for multiple linear regressions. An effect size of Cohens *f*^*2*^ = 0.024 was hereby determined for the smallest sample size (*N* = 553) when using one predictor and four total predictors with α = 0.05 and power (1-β) = 0.95. In short, an effect size slightly above the threshold of a small effect was detectable with sufficient power.

## Results

### Sample

Table 1 presents the sample’s sociodemographic and medical data. 153 of the patients were men (26.5%) and 424 were women (73.5%). The mean time since diagnosis was 11.9 months. Mean time since completion of acute treatment was 5.43 months *(SD 7.98)*, 478 patients *(82.4%)* had completed acute treatment (surgery, radiotherapy, chemotherapy, transplantation). The remaining 99 patients still were on hormone or antibody treatment, which is for some diagnosis like breast cancer according to guidelines necessary for up to 5 years after diagnosis [56]. Women with RCs were significantly older (*p* ≤ .001), more often partnered (*p* = .044), and had more children (*p* = .015) than women with NRCs. The most frequent diagnoses for females were: Breast Cancer (*n* = 150 patients; 35.5%), Hodgkin’s Lymphoma (*n* = 66; 15.6%) and Gynaecological Cancers (*n* = 51; 12.1%). In males, the most frequent diagnosis was Testicular Cancer (*n* = 50; 32.9%), followed by Hodgkin’s Lymphoma (*n* = 33; 21.7%), and Non-Hodgkin’s Lymphoma (*n* = 20; 13.2%). 3.3% of the sample was diagnosed with melanoma, a significantly lower rate than the nationwide incidence of 17.6% [8]. As expected, for women and men alike, NRCs and RCs differed in terms of the prescribed therapy regimes they underwent.

**Table 1:** Baseline characteristics of the sample

	Total (*N* = 577)	Women *n* = 424 (73.5%)					Men *n* = 153 (26.5%)				
		All	NRC *n* = 223	RC *n* = 201	*df*	*p*	All	NRC *n* = 103	RC *n* = 50	*df*	*p*
Age at diagnosis - Mean (*SD*)	29.30 (6.09)	29.09 (6.00)	27.72 (5.99)	32.32 (5.02)	422	≤.001***	27.76 (6.09)	27.26 (6.24)	28.78 (5.68)	151	.143
Months since diagnosis - Mean (*SD*)	11.89 (7.99)	11.78 (7.60)	11.85 (8.48)	11.69 (6.51)	406	.829	12.22 (9.01)	13.17 (8.97)	10.26 (8.87)	145	.061
	*N* (%)	*n* (%)	*n* (%)	*n* (%)			*n* (%)	*n* (%)	*n* (%)		
Partnership^a^	391 (67.8)	308 (73.2)	154 (69.1)	154 (77.8)	1	.044*	83 (54.2)	54 (52.4)	29 (58.0)	1	.516
Children	184 (31.9)	152 (35.8)	68 (30.5)	84 (41.8)	1	.015*	32 (21.1)	24 (23.5)	8 (16.0)	1	.285
Highest educational degree^b^					5	.509				5	
No educational degree	6 (1.0)	3 (0.7)	3 (1.3)	0			3 (2.0)	3 (3.0)	0		
Basic educational degree (< 10 years)	37 (6.5)	23 (5.5)	13 (5.8)	10 (5.2)			14 (9.2)	9 (8.8)	5 (10.0)		
Secondary educational degree (10 years)	90 (33.2)	128 (32.8)	74 (33.2)	64 (32.2)			52 (34.2)	34 (33.3)	18 (36.0)		
High school degree (> 10 years)	340 (59.3)	257 (61.1)	133 (59.6)	124 (62.6)			83 (54.5)	56 (54.9)	27 (54.0)		n/a
Housing/living conditions^c^					3	≤.001***				3	.200
Single	131 (22.7)	95 (23.6)	49 (23.0)	46 (12.2)			36 (24)	20 (19.8)	16 (32.7)		
Living with partner	298 (51.6)	238 (59.1)	110 (51.6)	128 (67.4)			60 (40)	41 (40.6)	19 (38.8)		
Living in parental household	92 (16.6)	51 (12.7)	40 (18.8)	11 (5.8)			41 (27.3)	32 (31.7)	9 (18.4)		
Shared community	32 (5.8)	19 (4.7)	14 (6.6)	5 (2.6)			13 (8.7)	8 (7.9)	5 (10.2)		0
Reproductive Cancer [C50-C57, C62]	251 (43.5)	201 (47.4)				n/a	50 (32.7)				
Non-Reproductive Cancer [All other C]	326 (56.5)	223 (52.6)					103 (67.3)				n/a
Cancer diagnosis						n/a					n/a
[C50] Breast	150 (26.0)	150 (35.4)					0				
[C81] Hodgkin’s Lymphoma	99 (17.2)	66 (15.6)					33 (21.7)				
[C51-C57] Gynecological	51 (8.8)	51 (12.0)					0				
[C62] Testicular	50 (8.7)	0					50 (32.7)				
[C82-C90] Non-Hodgkin’s Lymphoma	42 (7.3)	22 (5.2)					20 (13.2)				
Others	41 (7.1)	24 (5.7)					17 (11.1)				
[C91-C95] Haematological	38 (6.6)	20 (4.7)					16 (10.5)				
[C73] Thyroid	32 (5.5)	30 (7.1)					2 (1.3)				
[C15-C26] Gastrointestinal	29 (5.0)	21 (5.0)					8 (5.2)				
[C40-C41, C46-C49] Sarcoma	26 (4.5)	20 (4.7)					6 (3.9)				
[C43] Melanoma	19 (3.3)	18 (4.2)					1 (0.7)				
Therapies (multiple answers possible)^d^											
Chemotherapy^e^	443 (76.8)	310 (73.1)	152 (68.2)	158 (78.6)	2	.015*	133 (86.9)	91 (88.5)	42 (84)	2	.454
Surgery	427 (74.0)	325 (76.6)	139 (62.3)	186 (92.5)	2	≤.001***	102 (66.7)	54 (52.4)	48 (96)	2	≤.001***
Radio and nuclear therapy^e^	264 (45.8)	207 (48.8)	92 (41.3)	115 (57.2)	2	≤.001***	57 (37.3)	56 (54.4)	1 (2)	2	≤.001***
Stem Cell/bone marrow transplantation	33 (5.7)	23 (5.5)	21 (9.4)	2 (1.0)	2	≤.001***	10 (6.6)	9 (8.7)	1 (2)	2	.114

Males and females are listed separately and further broken down into groups of RCs and NRCs. t-tests and χ^2^ tests were performed between NRCs and RCs for each gender

Missing: ^a^3 (0.5%); ^b^4 (0.7%); ^c^24 (4.2%); ^d^Due to further validation there are deviations to the baseline medical therapies published in the study protocol [44]; ^e^including Radio-Chemotherpy; *n/a* not applicable

Table 2 presents the scores for the aspects *sexual satisfaction* (FLZ Sex), *sexual supportive care needs* (SCNS-SF34 Sex), *satisfaction with sexuality and partnership* (FLZ-M Sex/Partnership), and *changes in sexuality* (FLZ-MC Sex). No significant differences were found between male NRC and RC patients. Female RC patients reported significantly more sexual supportive care needs (*p* = .008) and more changes in their sexuality (*p* = .007). Women with RCs were also dissatisfied with their sexual responses significantly, one item of the FLZ-M Scale, more often than women with NRCs (*N = 412,* χ^2^ *6.3521, df 2, p* = .042).

**Table 2:** Equivalence tests (Two one sided tests - TOST)

Variable	All	NRC		RC		Test				TOST				
										Equivalence boundaries				
	*n*	*n*	*M (SD)*	*n*	*M (SD)*	*t*	*df*	*d*	*p*	Scale points	*d*	*t*	*df*	*p*
Men														
FLZ Sex	147	97	33.48 (8.59)	50	33.95 (7.84)	−0.319	145	0.06	.75	−4 to 4	−0.48 to 0.48	2.51	107.39	.007**
SCNS-SF34 Sex	153	103	21.12 (27.76)	50	19.67 (26.61)	0.307	151	0.05	.76	−16.67 to 16.67	−0.61 to 0.61	−3.27	100.92	≤.001***
FLZ-M Sex	147	98	3.30 (1.28)	49	3.31 (1.26)	−0.046	145	0.01	.96	−0.5 to 0.5	−0.39 to 0.39	−2.21	97.2	.015*
FLZ-MC Sex	151	102	2.28 (1.47)	49	2.22 (1.29)	0.243	149	−0.04	.81	−0.5 to 0.5	−0.35 to 0.35	1.87	106.75	.032*
Women														
FLZ Sex	416	219	30.58 (9.07)	197	28.81 (9.73)	1.918	414	0.19	.56	−4 to 4	−0.43 to 0.43	2.41	401.55	.008**
SCNS-SF34 Sex	424	223	26.46 (30.85)	201	35.03 (34.71)	−2.677^a^	402.435	0.26	.008**	−16.67 to 16.67	−0.51 to 0.51	2.53	402.44	.006**
FLZ-M Sex	408	214	3.14 (1.38)	194	3.02 (1.32)	0.928	406	0.09	.35	−0.5 to 0.5	−0.37 to 0.37	−2.84	404.55	.002**
FLZ-MC Sex	419	221	2.71 (1.57)	198	3.12 (1.48)	−2.693^a^	416.161	0.27	.007**	−0.5 to 0.5	−0.33 to 0.33	0.6	416.15	.273

* p < .05, ** p < .01, *** p < .001

*d = Cohen’s d,* ^*a*^ *= Welch’s t-test due to inhomogeneity of variance*

### Equivalence testing between NRCs and RCs

T-tests and, at follow-up, equivalence tests were performed to detect meaningful differences between NRC and RC patients in sexual satisfaction, changes in sexuality, sexual supportive care needs, and satisfaction with sexuality. T-tests compared NRC and RC patients divided by sex using the mean scores from the FLZ Sex scale, SCNS-SF34 Sex scale, FLZ-M Sex item, and the FLZ-MC Sex item. The two one-sided tests were performed using the equivalence boundaries explained in the method section. The detailed calculations are shown in Table 2. For females no significant differences between RCs and NRCs were detected on the sexual satisfaction (FLZSex) and satisfaction with sexuality (FLZ-M Sex) scales. In the succeeded equivalence tests (TOST), the changes in sexuality (FLZ-MC Sex) item was the only one for which no significant equivalence in score was found (*p* = .273). The other tested factors, sexual supportive care needs (SCNS-SF34 Sex), sexual satisfaction (FLZ Sex), and satisfaction with sexuality (FLZ-M Sex), were all within the previously determined equivalence boundaries. For the male patients, no significant differences were found (*t*-tests) and all scores were within the equivalence boundaries (TOST) in all of the tested categories: sexual satisfaction (FLZ Sex), sexual supportive care needs (SCNS-SF34 Sex), changes in sexuality (FLZ-MC Sex), and satisfaction with sexuality (FLZ-M Sex).

### NRC/RC as predictor of sexual satisfaction, supportive care needs, and changes in sexuality

To more comprehensively determine the influence of NRC vs. RC diagnosis status on sexual satisfaction, sexual supportive care needs, and changes in sexuality, we further performed multiple linear regressions using the known influencing factors partnership status, age, and gender as cofactors [48, 53, 54]. Normality, linearity, multicollinearity and independence of residuals were checked before without any concerns. These regression models are presented in Table 3. For the first model, all the established factors were entered. Female gender was significantly associated with each dependent variable (FLZ Sex, FLZ-M Sex, FLZ-MC Sex and SCNSC-SF34 Sex) in all of the regression models. Women were more likely than men to have lower sexual satisfaction scores (*b* = − 0.465, *p* ≤ .001) for the FLZ Sex scale and the FLZ-M Sex scale (*b* = − 0.229, *p* = .017), as well as to report greater changes in sexuality (*b* = 0.355, *p* ≤ .001) and more sexual supportive care needs (*b* = 7.798, *p* = .01). Partnership was significantly associated with greater sexual satisfaction (*b* = 0.485, *p* ≤ .001) on the FLZ Sex scale and the FLZ-M Sex scale (*b* = 0.488, *p* < .001), and greater changes in sexuality (*b* = 0.255, *p* = .008). Age was significantly associated with sexual satisfaction: each year of increased age corresponded with 0.016 SD drop in the FLZ Sex score (*p* = .013) and a 0.466 (*p* = .042) increase in sexual supportive care needs as measured by the SCNS SF-34 Sex scale. The factor reproductive cancer was added in the second model. None of the four regression models indicated that reproductive cancers significantly predict variances in the dependent variables when age, gender, and relationship are the cofactors in the regression analysis.

**Table 3:** Multiple linear regressions for FLZ Sex, SCNS-SF34 Sex, FLZ-M Sex, and FLZ-MC Sex scores

FLZ Sex *N* = 560	*b*	*SE*_*b*_	β	SCNS-SF34 Sex *N* = 574	*b*	*SE*_*b*_	β
Step 1				Step 1			
Constant	0.523	0.204		Constant	4.871	6.564	
Gender	−0.465	0.094	−.206***	Gender	7.798	3.021	.106*
Partnership	0.485	0.094	.224***	Partnership	5.250	2.992	.072
Age in years at diagnosis	−0.018	0.007	−.108*	Age in years at diagnosis	0.466	0.229	.084*
Step 2				Step 2			
Constant	0.490	0.204		Constant	6.476	6.643	
Gender	−0.457	0.094	−.202***	Gender	7.409	3.029	.101*
Partnership	0.484	0.094	.223***	Partnership	5.293	2.988	.073
Age in years at diagnosis	−0.015	0.007	−.092*	Age in years at diagnosis	0.357	0.239	.061
NRC/RC	−0.101	0.087	−.050	NRC/RC	4.225	2.796	.062
*R*^*2*^ = .079 for Step 1, *p* ≤ .001, *f*^*2*^ = 0.08 ∆ *R*^*2*^ = .002 for Step 2, *p* = .243				*R*^*2*^ = .036 for Step 1, *p ≤* .001, *f*^*2*^ = 0.03 ∆*R*^*2*^ = .004 for Step 2, *p* = .131			
FLZ-M Sex *N* = 553	*b*	*SE*_*b*_	β	FLZ-MC Sex *N* = 567	*b*	*SE*_*b*_	β
Step 1				Step 1			
Constant	0.076	0.210		Constant	−0.622	0.206	
Gender	−0.229	0.096	−.102*	Gender	0.355	0.095	.157***
Partnership	0.488	0.095	.226***	Partnership	0.251	0.094	.117**
Age in years at diagnosis	−0.008	0.007	−.05	Age in years at diagnosis	0.006	0.007	.039
Step 2				Step 2			
Constant	0.048	0.212		Constant	−0.563	0.208	
Gender	−0.222	0.096	−.098*	Gender	0.341	0.095	.151***
Partnership	0.488	0.095	.226***	Partnership	0.253	0.094	.118**
Age in years at diagnosis	−0.006	0.007	−.038	Age in years at diagnosis	0.002	0.007	.014
NRC/RC	−0.080	0.088	−.040	NRC/RC	0.159	0.087	.079
*R*^2^ =.051 for Step 1, *p* ≤ .001, *f*^*2*^ = 0.05, ∆*R*^2^ =.001 for Step 2, *p* = .363				*R*^2^ = .051 for Step 1*, p ≤* .001, *f*^*2*^ = 0.05, *∆R*^2^ =.006 for Step 2, *p =* .070			

* *p* < .05, ** *p* < .01, *** *p* < .001

*b* unstandardized regression weight, *β* standardized regression weight

Gender was coded 0 = male and 1 = female, partnership was coded single = 0 and partnered = 1

NRC was coded = 0 and RC was coded = 1

## Discussion

### General findings

The aim of this study was to investigate sexual satisfaction and supportive care needs in adolescent and young adult cancer patients and survivors, and to determine whether there are differences between NRC and RC patients in these areas. To our knowledge, this study is the first to focus on comparing NRCs and RCs in the AYA cancer patient/survivor population. There is also very little known about differences between NRCs and RCs in other cancer patient groups. Research on AYA cancer patients’ sexuality has mainly been conducted among patients with RCs, characterising them as a vulnerable group for sexual dissatisfaction. Little is known about NRC patients’ sexuality. Therefore, the evidence that AYA with RCs and NRCs have statistically significant equivalence in levels of sexual satisfaction is surprising. Once our scores were adjusted to account for gender, relationship status, and age, the fact of whether or not a patient’s cancer originated in a part of the body associated with sexual response did not predict satisfaction with sexuality (FLZ Sex and FLZ-M Sex), changes in sexuality (FLZ-MC Sex), or sexual supportive care needs (SCNS-SF34 Sex). Nevertheless we detected for women with RC compared to NRC, more changes in sexuality (FLZ-MC Sex) as well as higher supportive care needs (SCNS-SF34 Sex). In terms of satisfaction with sexuality (FLZ Sex and FLZ-M Sex) this difference was not found. In this study, 52% of the male NRC patients and 80% of the female RC patients reported at least a small change in their sex lives, which clearly indicates, that there is an impact of cancer and it’s treatment on sexuality. We also found, that partnered patients are more satisfied with their sexuality, men are more satisfied then women and older patients are less satisfied than younger patients.

Many threats to internal validity were excluded by the study design like historical events, testing, maturation diffusion. We looked at previous research to identify covariates and included them in our analyses. After all we adjusted for age, gender and status of partnership which are sociodemographic variables with virtual no possibility to be influenced by research design.

With clear inclusion and exclusion criteria we tried to improve external validity. A possible threat to external validity could be selection bias, as probably only higher motivated and not so sick patients are more willingly to participate voluntarily.

### Subsumption in context of the current state of research

A significant number of AYA cancer patients reported that the disease continues to impact their sex lives even after they have completed treatment. Bellizzi et al. [12] found that 45 to 63% of AYA patients with germ cell, lymphatic, and haematological cancers as well as sarcomas say their illness had a positive or negative impact on their sexuality. Champion et al. [36] reported that 41% of young breast cancer survivors experienced their sexual relationships to be changed for the worse. With percentages of patients reporting changes in their sex lives frequently above 50%, these findings clearly show the significant impact all types of cancer can have on a patient’s sexuality.

Previous research done in the general population has already shown that people in intimate relationships have more sexual intercourse [57] and that having sexual intercourse results in higher levels of sexual satisfaction [53, 58]. This is consistent with the present study’s finding that partnership predicted higher sexual satisfaction. Gender differences concerning levels of sexual satisfaction are also well known in AYAs [12, 48]. For the FLZ Sex scale, older age significantly predicted lower levels of sexual satisfaction in this study, something which is also true for the general population [45, 59]. It is therefore not surprising that these differences also exist in this sample even though the explained variance with these known influencers was low (0.02 < *f*^*2*^  <0.15) in all of the four realized regression analyses we performed.

Previous research has also indicated that patients with a wider range of diagnoses have said their sex lives were impacted by their illness. Jonker-Pool et al. [60] concluded in their comparison of male testicular (mean age: 36 years) and male malignant lymphoma patients (mean age: 42 years) that about one in three respondents from both groups suffered from one or more forms of sexual dysfunction. Perz et al. [41], who did a study with a sample of cancer survivors over 50 years old, found that participants with reproductive and non-reproductive cancers alike reported changes in their sexual activities and functioning that had occurred in connection with their illness. Flynn et al. [61] conducted qualitative research with a patient group older than the AYA age range (mean age: 50 years) who represented a variety of cancer diagnoses. They concluded that sexual satisfaction can be maintained even if sexual functioning is impaired. To our knowledge, no research has been done on whether this holds true for AYA cancer patients as well. One possible explanation for why sexual satisfaction and supportive care needs tend to be consistent across different types of diagnoses has been offered by Brotto et al. [62]. They studied gynaecological cancer patients and concluded that the psychological burden of cancer may be more salient than impairments that result from therapies or the cancer itself. Our results may support that theory in that, while significantly more women with RCs reported changes in their sexuality, at the same time, RC and NRC cancer patients reported statistically significant equivalent levels of sexual satisfaction. It stands to reason that impairment and satisfaction are, at least to a certain extent, decoupled. Ussher et al. [63] also found that even when different groups of patients (average age > 50 years and a variety of cancer diagnoses) experienced different amounts of change, their satisfaction levels still remained similar. While previous research indicated that psychological burden may be the main predictor of sexual satisfaction, our findings further suggest that there is no discernible difference between the amount of psychological burden AYA RC and NRC patients experience. Another important finding was that female RC patients, who reported greater changes in their sexuality when responding to the FLZ-MC question, also had scores for the SCNS-SF34 items *changes in sexuality* and *changes in sexual relationship* indicating that they need more support in these areas. Additionally, the fact that the female RC patients in our sample were more likely than the NRC patients to experience deterioration in their sexual responses but still reported similar levels of overall sexual satisfaction further underscores the theory that the psychological aspects of the experience of having cancer ultimately have a greater effect on sexual satisfaction than the physiological aspects do. At the same time, it is a known fact that surgery for breast or gynaecological cancer can affect a person’s ability to be sexually aroused via the breasts [64] and can result in reduced vaginal sensation [65]. In contrast to that, we found no differences in sexual responsiveness between testicular cancer and NRC patients, a very positive sign for current testicular cancer therapy regimes.

The significant difference in the sexual supportive care needs (SCNS-SF34 Sex) of women with NRCs in comparison to those with RCs (equivalence between ±2 raw points) raises the question of whether an effect size smaller than 2 raw points means that female RC patients should be treated as a more vulnerable group, or if this difference is too small to justify addressing this difference. As far as clinical practice is concerned, we recommend that doctors and other caregiving personnel address the sexual supportive care needs of all AYA patients as a matter of routine. After adjusting for gender, age, and relationship status, multiple linear regressions provided further confirmation that NRC vs. RC diagnoses do not significantly differ in terms of the explained variance in sexual supportive care needs those patients have (SCNS-SF34 Sex).

### Limitations

This study has certain limitations. First, the homogeneity of the study was limited due to the inclusion of patients with different types of diagnosis. This practice is however common and useful in AYA research. Second, the variety of diagnosis made it difficult to assess specific information concerning sexual dysfunction since addressing every separate entity individually would have required the use of a cancer-site specific instrument, the implementation of which would have been too complex to integrate into our study design. Hence, we could not draw definitive conclusions concerning specific connections between impairments and sexual satisfaction. Third, there were more missing values for the two FLZ Sex questions pertaining to partnership status than for any of the other items: 34 participants did not answer the questions. Of these, all but two were single. Presumably, single patients find it difficult to respond to these items or perhaps assume they do not apply to them even though they are in fact directed at both partnered and single respondents. Fourth, there is no clear rule for setting boundaries in equivalence testing [51] so we tried to establish meaningful boundaries based of the questionnaire we were using and degrees of difference we judged to be relevant. As far as Cohen’s “*d*” is concerned, boundaries ranged between (*d* = 0.31) and (*d* = 0.61), whereas the latter is a little higher than a medium effect size (*d* = 0.5). As such, potential effects equal to or bigger than this would have failed the test of equivalence. In combination with further tests such as multiple linear regressions, these equivalence boundaries clearly seem meaningful and consistent enough to use in clinical practice and psychological care for the purpose of identifying patients in need of support. Further discussion about meaningful equivalence boundaries and the resulting effect sizes should take place. Sixth, the sample size is an potential thread to generalizability, even our study is one of the larger sampled studies in the field of AYA research, a larger sample size for better generalizability would be desirable.

## Conclusions

So far, the question of how cancer affects patients’ sex lives has mainly been researched among RC patients [37]. It is increasingly apparent that these patients experience sexual impairments [36, 66] as well as decreased satisfaction [67, 68], and that these issues need to be addressed in counselling interventions [69]. Moreover, the findings of this study indicate that researchers and clinicians need to broaden their focus from RC patients to all AYA cancer patients concerning this topic. Almost 30% of the AYA patients we questioned said they need support in dealing with sexual problems and sexual dissatisfaction. Interestingly, there appeared to be little difference between RC and NRC patients in terms of their subjective experience of how satisfied they were with their sex lives after becoming ill with cancer. These results show that there is a clear need for clinical teams to address sexuality in AYA cancer patients as a matter of routine. An important step in this direction would be to integrate these topics into checklists such as the one presented by Hilgendorf et al. [9], which spells out questions to be explored with AYA patients, and is formulated for use before, during, and after treatment completion. Aubin et al. [70] have provided a succinct overview of how to assess the impact cancer is having on AYA patients’ sexuality in the clinical context.

Future research should focus on the impact non-reproductive cancers have on patients’ sexuality including: finding out what causes impairments; what the connections are between impairments and satisfaction; and developing strategies for dealing with these problems [35]. Further investigating the potential effects of the apparent disconnect between sexual impairment and sexual satisfaction could also be useful. To our knowledge, only one intervention study targeting sexuality in AYA cancer patients has been conducted so far, and it was done with a relatively small group *N* = 21. That study found that the intervention had a positive effect on participants’ knowledge regarding sexual issues, their body image, anxiety about sexual and romantic relationships, and their overall levels of psychological distress [71]. Further research with larger groups is needed, especially in light of the fact that the aforementioned study did not include any participants between the ages of 25 and 40 years old.

## Additional file

Additional file 1: This is the original “AYA-LE Questionnaire” which was designed by the “AYA-LE” study group. (PDF 567 kb).

## Data Availability

The datasets generated and analysed during the current study are not publicly available due to data protection regulations concerning patient information (which assures participants that the data will not be passed on to third parties), but are available from the corresponding author upon reasonable request.

## Abbreviations

AYA: Adolescent and young adult
NRC: Non-reproductive cancer
QoL: Quality of life
RC: Reproductive cancer
SPSS: Statistical Package for the Social Sciences
TOST: Two one-sided tests
