# Supplementary material for: Sexuality and cancer in adolescents and young adults - a comparison between reproductive cancer patients and patients with non-reproductive cancer
Source: BMC Cancer. 2019 Aug 22;19:828. doi: 10.1186/s12885-019-6009-2 (PMC6704507; doi:10.1186/s12885-019-6009-2)
Supplement: Supplementary file 1 — This is the original “AYA-LE Questionnaire” which was designed by the “AYA-LE” study group. (PDF 567 kb). [file 12885_2019_6009_MOESM1_ESM.pdf]

UNIVERSITÄT LEIPZIG
Medizinische Fakultät

Universitätsklinikum Leipzig
Medizin ist unsere Berufung.

Departement für Psychische Gesundheit
Abteilung für Medizinische Psychologie und Medizinische Soziologie

Fragebogen

Junge Erwachsene mit Krebs

Studie: Junge Erwachsene mit Krebs

„Lebenszufriedenheit, Versorgungssituation und Unterstützungsbedarf von Krebspatienten im jungen Erwachsenenalter“

**Bitte tragen Sie hier Ihren Patientencode ein !**
**Code** __ __ __ __ __ __

gefördert durch

Deutsche Krebshilfe
HELFEN. FORSCHEN. INFORMIEREN.

Sehr geehrte Dame, sehr geehrter Herr,

herzlichen Dank, dass Sie sich bereit erklärt haben, an unserer Befragung teilzunehmen. Bitte nehmen Sie sich die Zeit, dieses Fragebogenheft ungestört auszufüllen. Sie finden im Folgenden Fragen zu Ihrer Person, zu Ihrer Gesundheit und zu Ihrer psychosozialen Situation. **Wir bitten Sie darum, jede Frage zu lesen und zu beantworten.** Es gibt keine ‚richtigen' oder ‚falschen' Antworten. Falls eine Frage weniger auf Sie zutrifft oder es Ihnen einmal schwer fällt sich für eine Antwort zu entscheiden, kreuzen Sie bitte die Antwort an, die spontan am ehesten für Sie zutrifft. Bei einigen Fragen kann es zu Ähnlichkeiten mit vorherigen Fragen kommen, welche aus methodischen Gründen nicht zu vermeiden waren.

In der Mehrheit der Fälle werden Sie gebeten, Ihre Antworten durch Ankreuzen einer Antwortmöglichkeit kenntlich zu machen. ❑ weiblich ❑ männlich

Stellen, an denen wir Sie bitten, etwas aufzuschreiben, sind durch das Symbol eines Stifts gekennzeichnet. ✐ ___________________

Ihre Angaben werden **absolut vertraulich behandelt** und **ohne** Bezug auf Ihren Namen oder Ihre Person ausgewertet. Die Ergebnisse werden ausschließlich zu Forschungszwecken im Rahmen der Studie verwendet und nicht an Dritte weitergeleitet.

Werden Personenbezeichnungen aus Gründen der besseren Lesbarkeit lediglich in der männlichen Form verwendet, so schließt dies das jeweils weibliche Geschlecht mit ein.

Bei Fragen wenden Sie sich bitte direkt an uns:

Projektmitarbeiterinnen:
Katja Leuteritz (Diplom-Psychologin)
Tel.: 0341 9715405 E-Mail: Katja.Leuteritz@medizin.uni-leipzig.de

Annekathrin Sender (Diplom-Psychologin)
Tel.: 0341 9718804 E-Mail: Annekathrin.Sender@medizin.uni-leipzig.de

Projektleitung:
Dr. Kristina Geue und Prof. Dr. Yve Stöbel-Richter
Universitätsklinikum Leipzig AöR
Department für Psychische Gesundheit
Abteilung für Medizinische Psychologie und Medizinische Soziologie
Philipp-Rosenthal-Str. 55, 04103 Leipzig

Vielen Dank für Ihre Mitarbeit und Ihre Unterstützung!

A Persönliche Angaben

		Tag		Monat		Jahr			
A1	**Heutiges Datum:**					2	0		

A2

		Tag		Monat		Jahr	
a	**Geburtsdatum**						

b **Geschlecht** ❑0 männlich ❑1 weiblich

c **Staatsangehörigkeit** ❑0 deutsch ❑1 andere: ✎ ____________________

d1, d2 **Haben Sie zurzeit einen festen Partner?** ❑0 nein ❑1 ja, seit → Monat [ ][ ] Jahr [ ][ ]

↓

d3 **Leben Sie mit Ihrem Partner in einem Haushalt?** ❑1 ja ❑0 nein

B Fragen zur Krebserkrankung

B1 **Welche Krebsdiagnose wurde bei Ihnen gestellt?**

a Diagnose: ✎ ____

b Stadium:

❑0 Stadium 0	❑1 Stadium Ia	❑3 Stadium IIa	❑5 Stadium IIIa	❑7 Stadium IV
	❑2 Stadium Ib	❑4 Stadium IIb	❑6 Stadium IIIb	❑-8 weiß nicht

B2 **Wann wurde Ihre Krebserkrankung zum ersten Mal diagnostiziert?**

		Tag		Monat		Jahr	
a	**Diagnosedatum**						

B3 **Handelt es sich bei Ihrer aktuellen Krebserkrankung um** ❑0 eine Ersterkrankung ❑1 ein Rezidiv

B4 **Wurden bei Ihnen Metastasen diagnostiziert?** ❑0 nein ❑1 ja

B5 **Welche medizinischen Behandlungen haben Sie seit der Diagnosestellung bis zum heutigen Datum erhalten?** Geben Sie bitte ggf. auch die Anzahl der Zyklen/Therapien mit an!

		Durchführung: nein	Durchführung: ja, abgeschlossen	Durchführung: ja, läuft aktuell	Anzahl der abgeschlossenen Zyklen/Therapien
a1, a1z	Chemotherapie →	❑0	❑1	❑2	✎ ________
a2, a2z	Strahlen-Chemo-Therapie →	❑0	❑1	❑2	✎ ________
a3, a3z	Strahlentherapie →	❑0	❑1	❑2	✎ ________
a_4	Operation →	❑0	❑1	❑2	
a_5	Hormontherapie →	❑0	❑1	❑2	

→ *Fortsetzung nächste Seite*

		Durchführung			Anzahl der abgeschlossenen Zyklen/Therapien
		nein	ja, abgeschlossen	ja, läuft aktuell	
a_6	Knochenmarktransplantation →	❑0	❑1	❑2	
a_7	Stammzellentransplantation →	❑0	❑1	❑2	
a_8	Antikörpertherapie →	❑0	❑1	❑2	
a9, a9z	Sonstige ✎ ________________ →	❑0	❑1	❑2	✎ __________

B6	**Leiden Sie derzeit an weiteren schwerwiegenden körperlichen oder psychischen Erkrankungen?**
a	❑0 nein
b	❑1 ja → Wenn ja, woran? ✎ ________________________________
	________________________________
	________________________________

B7	**Hatte die onkologische Behandlung <u>Ihrer Einschätzung nach</u> die Heilung als Ziel?**	❑1 ja ❑0 nein ❑-8 weiß nicht

B8	**Haben Sie seit der onkologischen Diagnosestellung eine Rehabilitationsmaßnahme in Anspruch genommen?**
a	❑0 nein
	❑2 nein, aber ist geplant
b	❑1 ja → in welchem Zeitraum? ✎ von/vom ________________bis _______________

B9	**Seit wann ist Ihre Akutbehandlung (Operation, Chemotherapie, Strahlentherapie, Radio-Chemo-Therapie, Knochenmarks-/Stammzelltransplantation) abgeschlossen?**	Tag		Monat		Jahr	

C Fragen zur Erwerbstätigkeit

C1 a, b	**Sind Sie zurzeit krankgeschrieben?**	❑0 nein	❑1 ja, seit →	Monat		Jahr	

C2	**Wie viele Tage blieben Sie aufgrund der Krebserkrankung seit der Diagnosestellung bis heute der Arbeit fern?** (durch die Erkrankung selbst oder medizinische Untersuchungen, nicht durch Urlaub)				
	❑1	❑2	❑3	❑4	❑5
	überhaupt keinen	maximal 9 Tage	10 – 24 Tage	25 – 99 Tage	100 und mehr

C3	**Sind oder waren Sie vor Ihrer Krebserkrankung erwerbstätig?**<br>Unter Erwerbstätigkeit wird jede bezahlte bzw. mit einem Einkommen verbundene Tätigkeit verstanden, egal welchen zeitlichen Umfang sie hat.	
a	❑0 nein ↓	❑1 ja ↓

→ *Fortsetzung nächste Seite*

b,c1	**Nicht erwerbstätig: Sind/waren Sie…**	**Erwerbstätig: Sind/waren Sie…**
	❑1 in Berufsausbildung/Umschulung	❑1 Arbeiter
	❑2 Schüler	❑2 Angestellter
	❑3 Student	❑3 Beamter/Richter/Berufssoldat
	❑4 Hausfrau/-mann	❑4 Selbstständig
	❑5 in Elternzeit oder sonstiger Beurlaubung	❑5 Akademiker in freiem Beruf
	❑6 Bundesfreiwilligendienst (auch FSJ, FÖJ)	❑6 sonstiges: ✎ ____________
	❑7 (Früh-)Rentner	____________
	❑8 arbeitslos	____________
	❑9 sonstiges: ✎ ____________	↓
c2	____________	**Wie würden Sie Ihre Tätigkeit charakterisieren?**
	____________	❑1 ausführend unter Anleitung
		❑2 ausführend mit Entscheidungsbefugnissen
		❑3 führend bzw. leitend
c3		**Ist/war Ihr Arbeitsverhältnis befristet?**
		❑0 nein
		❑1 ja
c4		**Wie viele Stunden umfasst(e) Ihre wöchentliche Arbeitszeit laut Vertrag?**
		✎ ____________
c5		**Wie viele Stunden arbeiten/arbeiteten Sie tatsächlich?**
		✎ ____________

D Fragen zur Arbeitsbewältigung

D1 **Wie schätzen Sie Ihre derzeitige berufliche Leistungsfähigkeit im Allgemeinen ein?**
Wenn Sie Ihre maximale je erreichte Leistungsfähigkeit mit 10 Punkten bewerten:
Mit wie vielen Punkten würden Sie Ihre derzeitige berufliche Leistungsfähigkeit beschreiben?

☐	☐	☐	☐	☐	☐	☐	☐	☐	☐	☐
0	1	2	3	4	5	6	7	8	9	10
völlig arbeitsunfähig										maximal erreichte berufliche Leistungsfähigkeit

D2 **Wie schätzen Sie Ihre derzeitige berufliche Leistungsfähigkeit in Bezug auf die <u>körperlichen</u> Anforderungen ein?**

a

❑1	❑2	❑3	❑4	❑5
sehr schlecht	eher schlecht	mittelmäßig	eher gut	sehr gut

Falls Sie eingeschränkt leistungsfähig sind, ist dies...

b
❑1 wegen der Krebserkrankung/-behandlung
❑2 wegen einer anderen Krankheit, welcher? ✎ ____________

D3	**Wie schätzen Sie Ihre derzeitige berufliche Leistungsfähigkeit in Bezug auf die psychischen Anforderungen ein?**				
a	❑1 sehr schlecht	❑2 eher schlecht	❑3 mittelmäßig	❑4 eher gut	❑5 sehr gut
b	**Falls Sie eingeschränkt leistungsfähig sind, ist dies...**<br>❑1 wegen der Krebserkrankung/-behandlung<br>❑2 wegen einer anderen Krankheit, welcher? ✎ ______				

D4	**Meine Arbeit fordert mich:**		
	❑1 stärker körperlich als psychisch	❑2 stärker psychisch als körperlich	❑3 in gleichem Maße körperlich wie psychisch

D5	**Wie schätzen Sie die Wahrscheinlichkeit ein, dass Sie Ihrer derzeitigen (zuletzt ausgeübten) Arbeit in einem Jahr wieder nachgehen können?** Geben Sie bitte einen Wert von 0 bis 100% an.			
a	✎ ______ %			
b	**Und zwar im täglichen zeitlichen Umfang von:**			
	❑1 6 Stunden oder länger	❑2 3 bis 6 Stunden	❑3 weniger als 3 Stunden	❑-9 Keine Angabe

D6	**Beabsichtigen Sie, Ihre Arbeit wieder aufzunehmen?**			
a	❑1 ja	❑0 nein	❑2 ich arbeite bereits wieder	❑-8 weiß nicht
	↓	→ Weiter mit Frage **D8**		
b	**Wenn ja, in welchem Zeitraum nach Abschluss der Akutbehandlung? Innerhalb von:**			
	❑1 einem Monat	❑2 drei Monaten	❑3 sechs Monaten	❑4 zwölf Monaten
	❑5 nach zwölf Monaten	❑6 sobald als möglich	❑-8 weiß nicht	
c	↓<br>**Fühlen Sie sich bei dieser Entscheidung unter Druck gesetzt?**			
d	❑0 nein	❑1 ja →	**Falls ja, wodurch?** ✎ ______	

D7	**Sehen Sie Ihren Arbeitsplatz als gefährdet an?**			
a	❑0 nein			
b	❑1 ja → **wenn ja,** durch Ihre Erkrankung?	❑0 nein	❑1 ja	❑-8 kann ich nicht sagen

D8	**Wie oft hatten Sie in der vergangenen Woche ...**	immer	oft	manchmal	selten	nie/ fast nie
1	1. ... Konzentrationsprobleme?	❑1	❑2	❑3	❑4	❑5
2	2. ... Schwierigkeiten, Entscheidungen zu treffen?	❑1	❑2	❑3	❑4	❑5
3	3. ... Schwierigkeiten, sich zu erinnern?	❑1	❑2	❑3	❑4	❑5
4	4. ... Schwierigkeiten, klar zu denken?	❑1	❑2	❑3	❑4	❑5

E Fragen zur Befindlichkeit

E1	**Aktuelle Belastungen**

Bitte kreisen Sie am Thermometer rechts die Zahl ein (0-10) die am besten beschreibt, wie belastet Sie sich in der letzten Woche einschließlich heute gefühlt haben.

extrem belastet

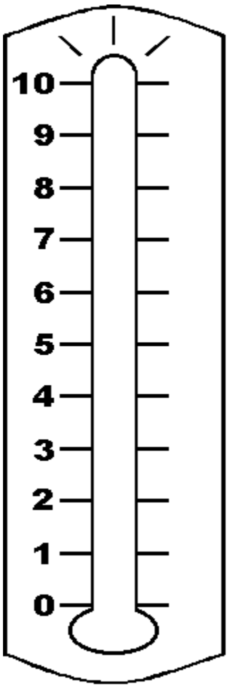

gar nicht belastet

E2<br>HADS	**Bitte kreuzen Sie die Antwortvorgabe an, die für Sie persönlich in der letzten Woche am ehesten zutraf!**		
a	Ich fühle mich angespannt oder überreizt.<br>❑1 = meistens<br>❑2 = oft<br>❑3 = von Zeit zu Zeit / gelegentlich<br>❑4 = überhaupt nicht	h	Ich fühle mich in meinen Aktivitäten gebremst.<br>❑1 = fast immer<br>❑2 = sehr oft<br>❑3 = manchmal<br>❑4 = überhaupt nicht
b	Ich kann mich heute noch so freuen wie früher.<br>❑1 = ganz genau so<br>❑2 = nicht ganz so sehr<br>❑3 = nur noch ein wenig<br>❑4 = kaum oder gar nicht	i	Ich habe manchmal ein ängstliches Gefühl in der Magengegend.<br>❑1 = überhaupt nicht<br>❑2 = gelegentlich<br>❑3 = ziemlich oft<br>❑4 = sehr oft
c	Mich überkommt eine ängstliche Vorahnung, dass etwas Schreckliches passieren könnte.<br>❑1 = ja, sehr stark<br>❑2 = ja, aber nicht allzu stark<br>❑3 = etwas, aber es macht mir keine Sorgen<br>❑4 = überhaupt nicht	j	Ich habe das Interesse an meiner äußeren Erscheinung verloren.<br>❑1 = Ja, stimmt genau.<br>❑2 = Ich kümmere mich nicht so sehr darum wie ich sollte.<br>❑3 = Möglicherweise kümmere ich mich zu wenig darum.<br>❑4 = Ich kümmere mich soviel darum wie immer.
d	Ich kann lachen und die lustige Seite der Dinge sehen.<br>❑1 = ja, so viel wie immer<br>❑2 = nicht mehr ganz so viel<br>❑3 = inzwischen viel weniger<br>❑4 = überhaupt nicht	k	Ich fühle mich rastlos, muss immer in Bewegung sein.<br>❑1 = ja, tatsächlich sehr<br>❑2 = ziemlich<br>❑3 = nicht sehr<br>❑4 = überhaupt nicht

e	Mir gehen beunruhigende Gedanken durch den Kopf.<br>❑1 = einen Großteil der Zeit<br>❑2 = verhältnismäßig oft<br>❑3 = von Zeit zu Zeit, aber nicht allzu oft<br>❑4 = nur gelegentlich/nie	l	Ich blicke mit Freude in die Zukunft.<br>❑1 = ja, sehr<br>❑2 = eher weniger als früher<br>❑3 = viel weniger als früher<br>❑4 = kaum bis gar nicht
f	Ich fühle mich glücklich.<br>❑1 = überhaupt nicht<br>❑2 = selten<br>❑3 = manchmal<br>❑4 = meistens	m	Mich überkommt plötzlich ein panikartiger Zustand.<br>❑1 = ja, tatsächlich sehr oft<br>❑2 = ziemlich oft<br>❑3 = nicht sehr oft<br>❑4 = überhaupt nicht
g	Ich kann behaglich dasitzen und mich entspannen.<br>❑1 = ja, natürlich<br>❑2 = gewöhnlich schon<br>❑3 = nicht oft<br>❑4 = überhaupt nicht	n	Ich kann mich an einem guten Buch, einer Radio- oder Fernsehsendung freuen.<br>❑1 = oft<br>❑2 = manchmal<br>❑3 = eher selten<br>❑4 = sehr selten

**E3**<br>PACIS	**Wie viel Mühe kostete es Sie <u>in der letzten Woche</u>, Ihre Erkrankung zu bewältigen?**<br>Bitte geben Sie einen Wert von 0 (= gar keine Mühe) bis 100 (= sehr viel Mühe) an:
	✎ ____________________

F Fragen zur Lebensqualität

**F1**	Jetzt stellen wir Ihnen einige Fragen zu Ihrem **<u>aktuellen</u>** allgemeinen Gesundheitsbefinden.				
EORTC QLQ-C30		überhaupt nicht	wenig	mäßig	sehr
1	Bereitet es Ihnen Schwierigkeiten, sich körperlich anzustrengen (z.B. eine schwere Einkaufstasche oder einen Koffer zu tragen)?	❑1	❑2	❑3	❑4
2	Bereitet es Ihnen Schwierigkeiten, einen <u>längeren</u> Spaziergang zu machen?	❑1	❑2	❑3	❑4
3	Bereitet es Ihnen Schwierigkeiten, eine <u>kurze</u> Strecke außer Haus zu gehen?	❑1	❑2	❑3	❑4
4	Müssen Sie tagsüber im Bett liegen oder in einem Sessel sitzen?	❑1	❑2	❑3	❑4
5	Brauchen Sie Hilfe beim Essen, Anziehen, Waschen oder Benutzen der Toilette?	❑1	❑2	❑3	❑4

**F2**	Die nun folgenden Fragen beziehen sich auf die **<u>letzte Woche</u>:**				
		überhaupt nicht	wenig	mäßig	sehr
1	Waren Sie bei Ihrer Arbeit oder bei anderen tagtäglichen Beschäftigungen eingeschränkt?	❑1	❑2	❑3	❑4
2	Waren Sie bei Ihren Hobbys oder bei anderen Freizeitbeschäftigungen eingeschränkt?	❑1	❑2	❑3	❑4
3	Waren Sie kurzatmig?	❑1	❑2	❑3	❑4
4	Hatten Sie Schmerzen?	❑1	❑2	❑3	❑4

		überhaupt nicht	wenig	mäßig	sehr
5	Mussten Sie sich ausruhen?	❑1	❑2	❑3	❑4
6	Hatten Sie Schlafstörungen?	❑1	❑2	❑3	❑4
7	Fühlten Sie sich schwach?	❑1	❑2	❑3	❑4
8	Hatten Sie Appetitmangel?	❑1	❑2	❑3	❑4
9	War Ihnen übel?	❑1	❑2	❑3	❑4
10	Haben Sie erbrochen?	❑1	❑2	❑3	❑4
11	Hatten Sie Verstopfung?	❑1	❑2	❑3	❑4
12	Hatten Sie Durchfall?	❑1	❑2	❑3	❑4
13	Waren Sie müde?	❑1	❑2	❑3	❑4
14	Fühlten Sie sich durch Schmerzen in Ihrem alltäglichen Leben beeinträchtigt?	❑1	❑2	❑3	❑4
15	Hatten Sie Schwierigkeiten, sich auf etwas zu konzentrieren, z.B. auf das Zeitunglesen oder das Fernsehen?	❑1	❑2	❑3	❑4
16	Fühlten Sie sich angespannt?	❑1	❑2	❑3	❑4
17	Haben Sie sich Sorgen gemacht?	❑1	❑2	❑3	❑4
18	Waren Sie reizbar?	❑1	❑2	❑3	❑4
19	Fühlten Sie sich niedergeschlagen?	❑1	❑2	❑3	❑4
20	Hatten Sie Schwierigkeiten, sich an Dinge zu erinnern?	❑1	❑2	❑3	❑4
21	Hat Ihr körperlicher Zustand oder Ihre medizinische Behandlung Ihr <u>Familienleben</u> beeinträchtigt?	❑1	❑2	❑3	❑4
22	Hat Ihr körperlicher Zustand oder Ihre medizinische Behandlung Ihr Zusammensein oder Ihre gemeinsamen Unternehmungen <u>mit anderen Menschen</u> beeinträchtigt?	❑1	❑2	❑3	❑4
23	Hat Ihr körperlicher Zustand oder Ihre medizinische Behandlung für Sie finanzielle Schwierigkeiten mit sich gebracht?	❑1	❑2	❑3	❑4
24<br>✎b	Hatten Sie andere Beschwerden? Bitte notieren Sie!<br>✎ ______________________<br>______________________	❑1	❑2	❑3	❑4

F3 Wie würden Sie insgesamt Ihren <u>GESUNDHEITSZUSTAND</u> während der **letzten Woche** einschätzen?

sehr schlecht **1** — **2** — **3** — **4** — **5** — **6** — **7** ausgezeichnet

F4 Wie würden Sie insgesamt Ihre <u>LEBENSQUALITÄT</u> während der **letzten Woche** einschätzen?

sehr schlecht **1** — **2** — **3** — **4** — **5** — **6** — **7** ausgezeichnet

F5 Wie würden Sie Ihre <u>LEBENSQUALITÄT</u> **vor der Diagnosestellung** einschätzen?

sehr schlecht **1** — **2** — **3** — **4** — **5** — **6** — **7** ausgezeichnet

F6 FA13	**Bitte beschreiben Sie, wie stark Sie die nachfolgenden Symptome oder Probleme <u>während der letzten Woche</u> empfunden haben.**				
		überhaupt nicht	wenig	mäßig	sehr
1	Hat es Ihnen an Energie gefehlt?	❑1	❑2	❑3	❑4
2	Fühlten Sie sich erschöpft?	❑1	❑2	❑3	❑4
3	Fühlten Sie sich verlangsamt?	❑1	❑2	❑3	❑4
4	Fühlten Sie sich tagsüber schläfrig?	❑1	❑2	❑3	❑4
5	Fiel es Ihnen schwer, Dinge in Angriff zu nehmen?	❑1	❑2	❑3	❑4
6	Fühlten Sie sich entmutigt?	❑1	❑2	❑3	❑4
7	Fühlten Sie sich hilflos?	❑1	❑2	❑3	❑4
8	Fühlten Sie sich frustriert?	❑1	❑2	❑3	❑4
9	Hatten Sie Schwierigkeiten, klar zu denken?	❑1	❑2	❑3	❑4
10	Fühlten Sie sich verwirrt?	❑1	❑2	❑3	❑4
11	Hatten Sie Schwierigkeiten, Dinge zu Ende zu bringen?	❑1	❑2	❑3	❑4
12	Hat Müdigkeit Ihre täglichen Aktivitäten beeinträchtigt?	❑1	❑2	❑3	❑4
13	Hatten Sie das Gefühl, dass die Menschen, die Ihnen nahe stehen, Ihre Müdigkeit nicht verstehen?	❑1	❑2	❑3	❑4

G Soziale Unterstützung

G1 SSUK	**Bei diesen Fragen geht es um Ihre Beziehungen zu wichtigen Menschen, also zum Partner, zu Familienangehörigen, Freunden und Bekannten, Kollegen und Nachbarn.**<br>Wir möchten erfahren, wie Sie diese Beziehungen erleben und einschätzen. Manchmal ist das Verhalten der anderen für uns sehr hilfreich, manchmal weniger, manchmal ist es auch belastend. Nachfolgend sind verschiedene Verhaltensweisen aufgeführt, die Menschen zeigen können, wenn jemand krank ist.<br>**Kreuzen Sie bitte an, wie oft sich eine oder mehrere der Ihnen nahestehenden Personen Ihnen gegenüber so verhalten haben.**					
	**Unter den Menschen, die Ihnen nahe stehen, gibt es jemanden, der ...**	nie	selten	manch-mal	häufig	immer
1	für Sie da ist, wenn Sie ihn brauchen.	❑1	❑2	❑3	❑4	❑5
2	zu viel Sorge oder Pessimismus in Bezug auf Ihre Erkrankung zeigt.	❑1	❑2	❑3	❑4	❑5
3	Sie aufmuntert oder tröstet.	❑1	❑2	❑3	❑4	❑5
4	Ihnen Informationen gibt oder Vorschläge macht, die Sie nicht hilfreich oder gar beunruhigend finden.	❑1	❑2	❑3	❑4	❑5
5	Ihnen das Gefühl gibt, dass Sie sich nicht um sich selbst kümmern können.	❑1	❑2	❑3	❑4	❑5
6	wichtige Entscheidungen mit Ihnen bespricht.	❑1	❑2	❑3	❑4	❑5
7	versucht, Sie auf eine unangenehme Art und Weise dazu zu bewegen, Ihre Form der Krankheitsbewältigung zu ändern.	❑1	❑2	❑3	❑4	❑5
8	einen Teil seiner Zeit oder Energie opfert, um etwas für Sie zu erledigen.	❑1	❑2	❑3	❑4	❑5

G2	Um die Versorgung von Krebspatienten zu verbessern, möchten wir erfahren, ob und in welchem Ausmaß Sie Unterstützung bei verschiedenen Aspekten Ihrer Krebserkrankung benötigen bzw. bereits erhalten haben.<br>**Bitte kreuzen Sie die Antwortmöglichkeit an, die am besten Ihr Bedürfnis nach Unterstützung beschreibt.**

scns		**<u>kein</u>** Bedürfnis nach Unterstützung vorhanden		Bedürfnis nach Unterstützung <u>vorhanden</u>		
	Während der **<u>letzten Woche</u>:**<br>Wie groß war Ihr Unterstützungsbedarf im Hinblick auf…	habe hier kein Problem	werde bereits unterstützt	gering	mittel	hoch
1	Schmerzen	❑1	❑2	❑3	❑4	❑5
2	Müdigkeit und Erschöpfung	❑1	❑2	❑3	❑4	❑5
3	Unwohlsein (meiste Zeit des Tages)	❑1	❑2	❑3	❑4	❑5
4	Arbeit im Haushalt	❑1	❑2	❑3	❑4	❑5
fer	die eigene Fruchtbarkeit (Fertilität)	❑1	❑2	❑3	❑4	❑5
5	Erledigung von Alltagsangelegenheiten	❑1	❑2	❑3	❑4	❑5
6	Angst	❑1	❑2	❑3	❑4	❑5
7	Niedergeschlagenheit oder Depression	❑1	❑2	❑3	❑4	❑5
8	Traurigkeit	❑1	❑2	❑3	❑4	❑5
9	Ängste vor dem Wiederauftreten/Fortschreiten der Krebserkrankung	❑1	❑2	❑3	❑4	❑5

scns		**<u>kein</u>** Bedürfnis nach Unterstützung vorhanden		Bedürfnis nach Unterstützung <u>vorhanden</u>		
	Während des **<u>letzten Monats</u>:**<br>Wie groß war Ihr Unterstützungsbedarf im Hinblick auf…	habe hier kein Problem	werde bereits unterstützt	gering	mittel	hoch
10	Sorgen darüber, das Ergebnis der Behandlung nicht kontrollieren zu können	❑1	❑2	❑3	❑4	❑5
11	Ungewissheit über die Zukunft	❑1	❑2	❑3	❑4	❑5
12	Erlangung eines Gefühls von Kontrolle über die Situation	❑1	❑2	❑3	❑4	❑5
13	Erhaltung einer positiven Sichtweise	❑1	❑2	❑3	❑4	❑5
14	Gedanken über Tod und Sterben	❑1	❑2	❑3	❑4	❑5
15	Veränderungen in der Sexualität	❑1	❑2	❑3	❑4	❑5
16	Veränderungen in der Partnerschaft	❑1	❑2	❑3	❑4	❑5
kiwu	Kinderwunsch	❑1	❑2	❑3	❑4	❑5
17	Gedanken über Sorgen Ihrer Angehörigen/Freunde	❑1	❑2	❑3	❑4	❑5
18	Freiheit bei der Wahl des behandelnden Arztes	❑1	❑2	❑3	❑4	❑5
19	Freiheit bei der Wahl des Krankenhauses	❑1	❑2	❑3	❑4	❑5
20	Verständnis und Wertschätzung der medizinischen Behandler (z.B. Ärzte, Pflegekräfte) für Ihre Gefühle	❑1	❑2	❑3	❑4	❑5
21	Behandlung körperlicher Probleme	❑1	❑2	❑3	❑4	❑5
22	Berücksichtigung Ihrer emotionalen Reaktionen während des Krankenhausaufenthaltes	❑1	❑2	❑3	❑4	❑5

→ *Fortsetzung nächste Seite*

scns	Während des **letzten Monats**: Wie groß war Ihr Unterstützungsbedarf im Hinblick auf…	**kein** Bedürfnis nach Unterstützung vorhanden: habe hier kein Problem	**kein** Bedürfnis nach Unterstützung vorhanden: werde bereits unterstützt	Bedürfnis nach Unterstützung vorhanden: gering	Bedürfnis nach Unterstützung vorhanden: mittel	Bedürfnis nach Unterstützung vorhanden: hoch
23	Erhalt von schriftlichen Informationen über zentrale Behandlungsaspekte	❑1	❑2	❑3	❑4	❑5
24	Erhalt von Informationen (schriftlich, Abbildungen, etc.) zum Umgang mit der Erkrankung und möglichen Behandlungsnebenwirkungen für zu Hause.	❑1	❑2	❑3	❑4	❑5
25	Erklärungen von Untersuchungsergebnissen	❑1	❑2	❑3	❑4	❑5
26	Erhalt von umfassenden Informationen zu Wirksamkeit und Nebenwirkungen der Behandlung vor Beginn	❑1	❑2	❑3	❑4	❑5
27	schnellstmögliche Information über Testergebnisse	❑1	❑2	❑3	❑4	❑5
28	schnellstmögliche Information über Tumorfreiheit bzw. Kontrolle der Tumorerkrankung	❑1	❑2	❑3	❑4	❑5
29	Information über mögliche Verhaltensänderungen, die das Wohlbefinden steigern	❑1	❑2	❑3	❑4	❑5
30	Zugang zu professioneller Beratung (z.B. Psychologe, Sozialarbeiter) für Sie oder Angehörige	❑1	❑2	❑3	❑4	❑5
31	Erhalt von Informationen über Sexualität	❑1	❑2	❑3	❑4	❑5
32	behandelt zu werden wie ein Mensch, nicht wie ein „Fall“	❑1	❑2	❑3	❑4	❑5
33	Behandlung in einem Krankenhaus mit freundlicher Ausstattung	❑1	❑2	❑3	❑4	❑5
34	fester Ansprechpartner für medizinische Fragen	❑1	❑2	❑3	❑4	❑5

H Fragen zur Lebenszufriedenheit

**H1** FLZM	**In welchem Maße hat es Veränderungen in den einzelnen Lebensbereichen seit der Diagnosestellung gegeben?**	gar nicht	wenig	etwas	ziemlich	sehr
1	Freunden/Bekannten	❑1	❑2	❑3	❑4	❑5
2	Freizeitgestaltung/Hobbies	❑1	❑2	❑3	❑4	❑5
3	Gesundheit	❑1	❑2	❑3	❑4	❑5
4	Einkommen/finanzielle Sicherheit	❑1	❑2	❑3	❑4	❑5
5	Beruf/Arbeit/Ausbildung	❑1	❑2	❑3	❑4	❑5
6	Wohnsituation	❑1	❑2	❑3	❑4	❑5
7	Familienleben	❑1	❑2	❑3	❑4	❑5
8	Kinder bzw. Familienplanung	❑1	❑2	❑3	❑4	❑5
9	Partnerschaft	❑1	❑2	❑3	❑4	❑5
10	Sexualität	❑1	❑2	❑3	❑4	❑5
11	Wie hoch schätzen Sie die Veränderungen in Ihrem Leben insgesamt ein, wenn Sie alle Aspekte zusammennehmen?	❑1	❑2	❑3	❑4	❑5

H2 LZM	**<u>Wie zufrieden</u> sind Sie gegenwärtig mit den einzelnen Lebensbereichen?**					
		unzu-frieden	eher unzufrieden	eher zufrieden	ziemlich zufrieden	sehr zufrieden
1	Freunden/Bekannten	❑1	❑2	❑3	❑4	❑5
2	Freizeitgestaltung/Hobbies	❑1	❑2	❑3	❑4	❑5
3	Gesundheit	❑1	❑2	❑3	❑4	❑5
4	Einkommen/finanzielle Sicherheit	❑1	❑2	❑3	❑4	❑5
5	Beruf/Arbeit/Ausbildung	❑1	❑2	❑3	❑4	❑5
6	Wohnsituation	❑1	❑2	❑3	❑4	❑5
7	Familienleben	❑1	❑2	❑3	❑4	❑5
8	Kinder bzw. Familienplanung	❑1	❑2	❑3	❑4	❑5
9	Partnerschaft	❑1	❑2	❑3	❑4	❑5
10	Sexualität	❑1	❑2	❑3	❑4	❑5
11	Wie zufrieden sind Sie mit Ihrem Leben insgesamt, wenn Sie alle Aspekte zusammennehmen?	❑1	❑2	❑3	❑4	❑5

H3	**War bei Ihnen zum Zeitpunkt der Krebsdiagnose die Familienplanung (eigener Kinderwunsch) bereits abgeschlossen?**	
	❑0 nein → weiter mit **H4**	❑1 ja → weiter mit **H6**

H4	**Wie stark ist Ihr momentaner Kinderwunsch?**				
	sehr gering				sehr stark
	❑1	❑2	❑3	❑4	❑5

H5 Wurden Maßnahmen zur Erhaltung der Fruchtbarkeit bei Ihnen durchgeführt (Fertiprotektion)?

a ❑0 nein ❑1 ja → wenn ja, welche?

b
- ❑1 Kryokonservierung Sperma
- ❑2 Kryokonservierung Eizellen
- ❑3 Kryokonservierung Ovargewebe
- ❑4 GnRH-Analoga
- ❑5 Sonstiges ✎ ____________________

H6 FLZ

Im Folgenden geht es um die Bereiche Sexualität und Partnerschaft. Kreuzen Sie bitte die Antwortmöglichkeit an, **die am ehesten Ihrer Zufriedenheit** entspricht.

Bitte beantworten Sie **alle Fragen**, auch diejenigen, die scheinbar nicht auf Sie zutreffen: Wenn Sie zum Beispiel keinen Partner haben, können Sie bei den Fragen nach der „Partnerschaft" trotzdem angeben, wie zufrieden Sie mit der derzeitigen Situation (ohne Partner) sind.

		sehr unzu-frieden	unzu-frieden	eher unzu-frieden	weder/ noch	eher zu-frieden	zu-frieden	sehr zu-frieden
1	Mit meiner körperlichen Attraktivität bin ich…	❑1	❑2	❑3	❑4	❑5	❑6	❑7
2	Mit meiner sexuellen Leistungs-fähigkeit bin ich…	❑1	❑2	❑3	❑4	❑5	❑6	❑7

→ *Fortsetzung nächste Seite*

		sehr unzu-frieden	unzu-frieden	eher unzu-frieden	weder/ noch	eher zu-frieden	zu-frieden	sehr zu-frieden
3	Mit der Häufigkeit meiner sexuellen Kontakte bin ich…	❑1	❑2	❑3	❑4	❑5	❑6	❑7
4	Mit der Häufigkeit, mit der meine (Ehe-) Partner(in) sich mir körperlich zuwendet (streichelt, berührt) bin ich…	❑1	❑2	❑3	❑4	❑5	❑6	❑7
5	Mit meinen sexuellen Reaktionen bin ich…	❑1	❑2	❑3	❑4	❑5	❑6	❑7
6	Wenn ich daran denke, wie unbefangen ich über den sexuellen Bereich sprechen kann, bin ich…	❑1	❑2	❑3	❑4	❑5	❑6	❑7
7	Wenn ich daran denke, inwiefern mein Partner und ich in der Sexualität harmonisieren, bin ich…	❑1	❑2	❑3	❑4	❑5	❑6	❑7

K Fragen zur Lebenseinstellung

K1	**Geben Sie an, wie sehr die folgenden Aussagen ganz allgemein auf Sie zutreffen.**							
	**Ganz allgemein betrachtet, tue ich Dinge …**	über-haupt nicht zutref-fend	sehr wenig zutref-fend	etwas zutref-fend	einiger-maßen zutref-fend	stark zutref-fend	sehr stark zutref-fend	völlig zutref-fend
1	… weil ich bestimmte Menschen nicht enttäuschen möchte.	❑1	❑2	❑3	❑4	❑5	❑6	❑7
2	... um zu dem Menschen zu werden, der ich gerne werden möchte.	❑1	❑2	❑3	❑4	❑5	❑6	❑7
3	… weil sie darstellen, wer ich bin.	❑1	❑2	❑3	❑4	❑5	❑6	❑7
4	... auch, wenn ich in ihnen keinen Nutzen sehe.	❑1	❑2	❑3	❑4	❑5	❑6	❑7
5	… weil ich möchte, dass andere Menschen ein positives Bild von mir haben.	❑1	❑2	❑3	❑4	❑5	❑6	❑7
6	… weil ich sie als Mittel gewählt habe, um meine Ziele zu erreichen.	❑1	❑2	❑3	❑4	❑5	❑6	❑7
7	... weil es Spaß macht, Neues zu lernen.	❑1	❑2	❑3	❑4	❑5	❑6	❑7
8	... weil ich mich schuldig fühlen würde, wenn ich sie nicht täte.	❑1	❑2	❑3	❑4	❑5	❑6	❑7
9	... weil sie mit meinen wesentlichen Ansichten übereinstimmen.	❑1	❑2	❑3	❑4	❑5	❑6	❑7
10	… auch, wenn es keinen Unterschied macht, ob ich sie tue oder nicht.	❑1	❑2	❑3	❑4	❑5	❑6	❑7
11	... wegen der angenehmen Gefühle, die ich empfinde, während ich die Dinge tue.	❑1	❑2	❑3	❑4	❑5	❑6	❑7
12	… um anderen zu zeigen, was ich kann.	❑1	❑2	❑3	❑4	❑5	❑6	❑7

→ *Fortsetzung nächste Seite*

	Ganz allgemein betrachtet, tue ich Dinge …	über-haupt nicht zutref-fend	sehr wenig zutref-fend	etwas zutref-fend	einiger-maßen zutref-fend	stark zutref-fend	sehr stark zutref-fend	völlig zutref-fend
13	… weil ich mich dazu zwinge.	❑1	❑2	❑3	❑4	❑5	❑6	❑7
14	… wegen der Zufriedenheit, die ich empfinde, während ich versuche etwas besonders gut zu machen.	❑1	❑2	❑3	❑4	❑5	❑6	❑7
15	… auch, wenn ich keinen guten Grund habe, sie zu tun.	❑1	❑2	❑3	❑4	❑5	❑6	❑7
16	… weil ich mich für das einsetze, was mir wichtig ist.	❑1	❑2	❑3	❑4	❑5	❑6	❑7
17	… weil ich verärgert über mich wäre, wenn ich sie nicht täte.	❑1	❑2	❑3	❑4	❑5	❑6	❑7
18	... weil sie darstellen, worauf ich im Leben am meisten Wert lege.	❑1	❑2	❑3	❑4	❑5	❑6	❑7

L Fragen zum Gesundheitsverhalten

L1 **Bitte geben Sie für jede der folgenden Verhaltensweisen bzw. Tätigkeiten an, wie häufig Sie diese tun.**

		nie	selten	häufig	fast immer	immer
1	regelmäßige Gesundheitsuntersuchungen (z.B. Vorsorgeuntersuchungen, Funktionsprüfungen von Organen)	❑1	❑2	❑3	❑4	❑5
2	bei Wahrnehmung von Symptomen einen Arzt aufsuchen (Vermeiden notwendige Termine aufzuschieben)	❑1	❑2	❑3	❑4	❑5
3	sich an therapeutische Anordnungen von Ärzten (etc.) halten (z.B. Medikamenteneinnahme o.ä.)	❑1	❑2	❑3	❑4	❑5
4	sich richtig und ausgewogenen ernähren (vitamin-, mineralien-, ballaststoffreich)	❑1	❑2	❑3	❑4	❑5
5	Verzehr von Süßigkeiten	❑1	❑2	❑3	❑4	❑5
6	Verzehr von fettreichen Speisen	❑1	❑2	❑3	❑4	❑5
7	sportliches Verhalten und Bewegung (für regelmäßige Körperbewegung sorgen, z.B. Sport, Spaziergänge)	❑1	❑2	❑3	❑4	❑5
8	Verbrauch von Kaffee	❑1	❑2	❑3	❑4	❑5
9	Verbrauch „weicher“ Alkoholika (Bier, Wein)	❑1	❑2	❑3	❑4	❑5
10	Verbrauch „harter“ Alkoholika (Schnaps etc.)	❑1	❑2	❑3	❑4	❑5
11	Nikotinkonsum (Rauchen von Zigaretten, Zigarren, Pfeifen etc.)	❑1	❑2	❑3	❑4	❑5
12	im Auto den Sicherheitsgurt anlegen	❑1	❑2	❑3	❑4	❑5
13	bewusst leben (subjektiv sinnvolle Dinge tun)	❑1	❑2	❑3	❑4	❑5
14	sich im Straßenverkehr rücksichtsvoll verhalten (z.B. Geschwindigkeitsbegrenzungen einhalten, hohe Geschwindigkeiten vermeiden)	❑1	❑2	❑3	❑4	❑5
15	aktiv und schöpferisch sein	❑1	❑2	❑3	❑4	❑5

M Fragen zur psychosozialen Versorgungssituation

M1 Bitte geben Sie für die nachfolgend genannten psychosozialen Versorgungsangebote an, ob diese Ihnen **<u>während Ihrer stationären Krankenhausaufenthalte</u>** <u>(nicht Rehabilitationsmaßnahme)</u> angeboten wurden, welche Sie in Anspruch genommen haben und wie hilfreich es für Sie war!

	**wurde angeboten/ war vorhanden** M1a1	**Inanspruchnahme** M1a2	**Häufigkeit** M1a3	**Wie hilfreich war das Angebot für Sie?** M1a4
**a) psychologische Beratung** (Psychoonkologe, Psychologe)	❑1 ja ➡<br>❑0 nein<br>❑-8 weiß nicht	❑1 ja ➡<br><br>❑0 nein ➡	❑1 1x<br>❑2 2-3x<br>❑3 >3x	❑1 sehr ❑4 wenig<br>❑2 ziemlich ❑5 gar nicht<br>❑3 etwas

Gründe: (Mehrfachnennungen möglich) M1a5

❑1 kein Bedarf ❑2 kein Interesse

❑3 zeitlich unpassend

❑4 körperlich nicht in der Lage

❑5 psychisch nicht in der Lage

sonstiges: ✎ ______________________________

	**wurde angeboten/ war vorhanden** M1b1	**Inanspruchnahme** M1b2	**Häufigkeit** M1b3	**Wie hilfreich war das Angebot für Sie?** M1b4
**b) sozialrechtliche Beratung** (Sozialarbeiter)	❑1 ja ➡<br>❑0 nein<br>❑-8 weiß nicht	❑1 ja ➡<br><br>❑0 nein ➡	❑1 1x<br>❑2 2-3x<br>❑3 >3x	❑1 sehr ❑4 wenig<br>❑2 ziemlich ❑5 gar nicht<br>❑3 etwas

Gründe: (Mehrfachnennungen möglich) M1b5

❑1 kein Bedarf ❑2 kein Interesse

❑3 zeitlich unpassend

❑4 körperlich nicht in der Lage

❑5 psychisch nicht in der Lage

sonstiges: ✎ ______________________________

	**wurde angeboten/ war vorhanden** M1c1	**Inanspruchnahme** M1c2	**Häufigkeit** M1c3	**Wie hilfreich war das Angebot für Sie?** M1c4
**c) Anderes psychosoziales Angebot** (z.B. Kreativ-, bewegungs-therapeutische- oder Gesprächs-angebote etc.)<br><br>Name des Angebotes M1c0<br><br>✎ ____________	❑1 ja ➡<br>❑0 nein<br>❑-8 weiß nicht	❑1 ja ➡<br><br>❑0 nein ➡	❑1 1x<br>❑2 2-3x<br>❑3 >3x	❑1 sehr ❑4 wenig<br>❑2 ziemlich ❑5 gar nicht<br>❑3 etwas

Gründe: (Mehrfachnennungen möglich) M1c5

❑1 kein Bedarf ❑2 kein Interesse

❑3 zeitlich unpassend

❑4 körperlich nicht in der Lage

❑5 psychisch nicht in der Lage

sonstiges: ✎ ______________________________

M2	**Wie wurden Sie auf psychoonkologische Angebote <u>während Ihrer stationären Aufenthalte</u> aufmerksam?** *(Mehrfachnennungen möglich)*
	❑1 Onkologen<br>❑2 anderes Krankenhauspersonal (Psychologen, Sozialarbeiter, Krankenschwester etc.)<br>❑3 andere Betroffene<br>❑4 Familie/Angehörige/Freunde<br>❑5 Aushänge/Flyer/Broschüren<br>❑6 Sonstiges: 🖉 ____________________<br>❑7 Ich bin über kein psychoonkologisches Angebot informiert worden.

M3	**Mit welchem Anliegen würden Sie persönlich momentan ein psychosoziales Angebot in Anspruch nehmen?**
	1. ____________________<br>2. ____________________<br>3. ____________________

M4	**Welche Themen sollten in der psychosozialen Versorgung von jungen Krebspatienten Ihrer Meinung nach generell berücksichtigt werden? Bitte nennen Sie die 5 wichtigsten!**
	1. ____________________<br>2. ____________________<br>3. ____________________<br>4. ____________________<br>5. ____________________

M5	**Welche der genannten psychoonkologischen Angebote würden Sie gegenwärtig in Anspruch nehmen und in welcher Form?**	
a1	**a) psychologische Beratung**	❑1 ja ❑2 vielleicht ❑0 nein
	wenn ja: *(Mehrfachnennungen möglich)*	
a2, a3	**In welcher Form?**<br>❑1 als Einzelberatung<br>❑2 als Gruppenangebote mit Gleichaltrigen<br>❑3 als Gruppenangebote (altersgemischt)<br>❑4 als Paarberatung ❑5 als Familienberatung	**In welcher Kontaktform?**<br>❑1 persönlich<br>❑2 telefonisch<br>❑3 online

b1	**b) soziale/sozialrechtliche Beratung**	❑1 ja ❑2 vielleicht ❑0 nein
	wenn ja: *(Mehrfachnennungen möglich)*	
b2, b3	**In welcher Form?**<br>❑1 als Einzelberatung<br>❑2 als Gruppenangebote mit Gleichaltrigen<br>❑3 als Gruppenangebote (altersgemischt)<br>❑4 mit Partner ❑5 als Familienberatung	**In welcher Kontaktform?**<br>❑1 persönlich<br>❑2 telefonisch<br>❑3 online

c1	**c) Patientenschulung**	❑1 ja ❑2 vielleicht ❑0 nein
	wenn ja: *(Mehrfachnennungen möglich)*	
c2,c3	**In welcher Form?**<br>❑1 als Einzelschulung<br>❑2 als Gruppenangebote mit Gleichaltrigen<br>❑3 als Gruppenangebote (altersgemischt)<br>❑4 mit Partner	**Zu welchen Themen?**<br>1. ____________<br>2. ____________<br>3. ____________

d1	**d) kreative Therapieangebote** (Musik, Kunst etc.)	❑1 ja ❑2 vielleicht ❑0 nein
	wenn ja: *(Mehrfachnennungen möglich)*	
d2	**In welcher Form?**<br>❑1 als Einzelangebot<br>❑2 als Gruppenangebote mit Gleichaltrigen<br>❑3 als Gruppenangebote (altersgemischt)<br>❑4 mit Partner	

e1	**e) Bewegungstherapeutische Angebote** (Tanz, Sport etc.)	❑1 ja ❑2 vielleicht ❑0 nein
	wenn ja: *(Mehrfachnennungen möglich)*	
e2	**In welcher Form?**<br>❑1 als Einzelangebot<br>❑2 als Gruppenangebote mit Gleichaltrigen<br>❑3 als Gruppenangebote (altersgemischt)<br>❑4 mit Partner	

f1	**f) Entspannungsverfahren** (Yoga, QiGong, Autogenes Training etc.)	❑1 ja ❑2 vielleicht ❑0 nein
	wenn ja: *(Mehrfachnennungen möglich)*	
f2	**In welcher Form?**<br>❑1 als Einzelangebot<br>❑2 als Gruppenangebote mit Gleichaltrigen<br>❑3 als Gruppenangebote (altersgemischt)<br>❑4 mit Partner	

**M6**	**Wie wichtig sind Ihnen die folgenden psychosozialen Angebote?**					
		sehr	ziemlich	etwas	wenig	gar nicht
1	psychologische Beratung	❑1	❑2	❑3	❑4	❑5
2	soziale/sozialrechtliche Beratung	❑1	❑2	❑3	❑4	❑5
3	Patientenschulung	❑1	❑2	❑3	❑4	❑5
4	kreative Therapieangebote (Musik, Kunst etc.)	❑1	❑2	❑3	❑4	❑5
5	Bewegungstherapeutische Angebote (Tanz, Sport etc.)	❑1	❑2	❑3	❑4	❑5
6	Entspannungsverfahren (Yoga, QiGong, Autogenes Training etc.)	❑1	❑2	❑3	❑4	❑5

M7	**Welche psychosozialen Versorgungsangebote haben Sie während Ihrer stationären Aufenthalte vermisst?**
	1. ______________________
	2. ______________________
	3. ______________________
	4. ______________________
	5. ______________________

M8	**Wie häufig würden Sie generell ein psychosoziales Angebot in Anspruch nehmen?**		
	❑1 einmalig	❑2 jährlich	❑3 halbjährlich
	❑4 monatlich	❑5 wöchentlich	❑6 gar nicht

M9	**Wie zufrieden sind Sie insgesamt mit der psychosozialen Versorgung?**				
	❑1 sehr	❑2 ziemlich	❑3 etwas	❑4 wenig	❑5 gar nicht

Abschließend haben wir noch einige wenige Fragen zu Ihrer Person.

N1	**Welchen Familienstand haben Sie?**				
	ledig	verheiratet (mit dem Ehepartner zusammenlebend)	verheiratet (in Trennung lebend)	geschieden	verwitwet
	❑1	❑2	❑3	❑4	❑5

N2	**Wie ist Ihre derzeitige Wohnsituation?** (Kinder unberücksichtigt)<br>**Ich lebe…**	
	❑1 …allein	❑3 …in einer Wohngemeinschaft
	❑2 ...mit meinem Partner zusammen	❑4 …bei meinen Eltern
	❑5 Sonstiges, und zwar: ✎ ______________________	

N3<br>a<br>b<br>c	**Haben Sie eigene Kinder?**<br>(einschließlich Pflege- oder Stiefkinder)	❑0 nein<br>❑1 ja, ich habe ✎_______ Kinder (Anzahl)<br>Wie viele davon leben in Ihrem Haushalt? ✎_______

N4	**Wie viele Personen leben insgesamt in Ihrem Haushalt, Sie selbst eingeschlossen?**<br>Zählen Sie dabei bitte auch Kinder mit und tragen Sie die Anzahl ein
a, b	Insgesamt ✎________ Personen, davon sind ✎____________ unter 18 Jahre alt

N5	**Seit wann leben Sie in Deutschland?**
a	Seit meiner Geburt → ❑1
b	Seit (Jahreszahl) → ______________

N6	**In welchem Bundesland haben Sie bisher überwiegend gelebt?**	______________________________

N7	**Welchen höchsten allgemeinbildenden Schulabschluss haben Sie?**
	❑1 noch Schüler
	❑2 von der Schule abgegangen ohne Haupt-/Volksschulabschluss
	❑3 Haupt-/Volksschulabschluss
	❑4 Mittlere Reife / Realschulabschluss / Abschluss der POS
	❑5 Fachhochschulreife
	❑6 allgemeine oder fachgebundene Hochschulreife / Abitur (Gymnasium, EOS)
	❑7 Sonstiges, und zwar: ______________________________

N8	**Welches ist Ihr höchster beruflicher Abschluss?**
	❑1 kein beruflicher Abschluss und nicht in Ausbildung
	❑2 noch in beruflicher Ausbildung (Auszubildende/r, Student/in)
	❑3 abgeschlossene Lehre (beruflich-betriebliche Ausbildung)
	❑4 Handelsschule/Berufsfachschule (beruflich-schulische Ausbildung)
	❑5 Abschluss an Fachschule, Meister-, Technikerschule, Berufs- oder Fachakademie
	❑6 Fachhochschulabschluss
	❑7 Hochschulabschluss
	❑8 Promotion
	❑9 Sonstiges, und zwar: ______________________________

N9	**Wie hoch ist das monatliche Nettoeinkommen aller Haushaltsmitglieder insgesamt?** Nettoeinkommen: Die Summe aus Lohn/Gehalt/Einkommen usw. (nach Abzug von Steuern und Sozialabgaben)		
	❑1 unter 500 €	❑6 2.500 € bis unter 3.000 €	
	❑2 500 € bis unter 1.000 €	❑7 3.000 € bis unter 4.000 €	
	❑3 1.000 € bis unter 1.500 €	❑8 4.000 € bis unter 5.000 €	
	❑4 1.500 € bis unter 2.000 €	❑9 5.000 € bis unter 7.000 €	❑-8 weiß nicht
	❑5 2.000 € bis unter 2.500 €	❑10 7.000 € und mehr	❑-9 keine Angabe

N10	**Gehören Sie einer Glaubensgemeinschaft/Religion an?**	❑0 nein ❑1 ja
a		↓ Welcher?
b		______________________________

O1	**Wären Sie zu einem vertiefenden (Telefon-) Interview bereit?**	❑0 nein ❑1 ja

Bitte überprüfen Sie noch einmal, ob Sie alle Fragen beantwortet haben!

Vielen Dank für das Ausfüllen des Fragebogens!

Gibt es Ihrer Meinung nach Fragen/Themen, die in dieser Befragung unberücksichtigt blieben?
Weitere Ergänzungen oder Anmerkungen können Sie hier notieren:

O2
